# Auxin-Mediated Transcriptional System with a Minimal Set of Components Is Critical for Morphogenesis through the Life Cycle in *Marchantia polymorpha*

**DOI:** 10.1371/journal.pgen.1005084

**Published:** 2015-05-28

**Authors:** Hirotaka Kato, Kimitsune Ishizaki, Masaru Kouno, Makoto Shirakawa, John L. Bowman, Ryuichi Nishihama, Takayuki Kohchi

**Affiliations:** 1 Graduate School of Biostudies, Kyoto University, Kyoto, Japan; 2 Graduate School of Science, Kobe University, Kobe, Japan; 3 School of Biological Sciences, Monash University, Melbourne, Victoria, Australia; 4 Section of Plant Biology, University of California, Davis, Davis, California, United States of America; Harvard University, UNITED STATES

## Abstract

The plant hormone auxin regulates many aspects of plant growth and development. Recent progress in Arabidopsis provided a scheme that auxin receptors, TIR1/AFBs, target transcriptional co-repressors, AUX/IAAs, for degradation, allowing ARFs to regulate transcription of auxin responsive genes. The mechanism of auxin-mediated transcriptional regulation is considered to have evolved around the time plants adapted to land. However, little is known about the role of auxin-mediated transcription in basal land plant lineages. We focused on the liverwort *Marchantia polymorpha*, which belongs to the earliest diverging lineage of land plants. *M. polymorpha* has only a single *TIR1/AFB* (*MpTIR1*), a single *AUX/IAA* (*MpIAA*), and three *ARFs* (*MpARF1*, *MpARF2*, and *MpARF3*) in the genome. Expression of a dominant allele of MpIAA with mutations in its putative degron sequence conferred an auxin resistant phenotype and repressed auxin-dependent expression of the auxin response reporter *_pro_GH3*:*GUS*. We next established a system for DEX-inducible auxin-response repression by expressing the putatively stabilized MpIAA protein fused with the glucocorticoid receptor domain (MpIAA^mDII^-GR). Repression of auxin responses in *_pro_MpIAA:MpIAA^mDII^-GR* plants caused severe defects in various developmental processes, including gemmaling development, dorsiventrality, organogenesis, and tropic responses. Transient transactivation assays showed that the three MpARFs had different transcriptional activities, each corresponding to their phylogenetic classifications. Moreover, MpIAA and MpARF proteins interacted with each other with different affinities. This study provides evidence that pleiotropic auxin responses can be achieved by a minimal set of auxin signaling factors and suggests that the transcriptional regulation mediated by TIR1/AFB, AUX/IAA, and three types of ARFs might have been a key invention to establish body plans of land plants. We propose that *M*. *polymorpha* is a good model to investigate the principles and the evolution of auxin-mediated transcriptional regulation and its roles in land plant morphogenesis.

## Author Summary

Auxin is an important plant hormone which regulates many aspects of plant growth and development, such as embryogenesis and directional growth in response to light or gravity. Recent molecular genetics advances in angiosperms revealed that transcriptional regulation is critical for auxin response. Although auxin response was also observed in charophytes, a class of green algae related to land plants, and bryophytes, little is known how plants acquired auxin signaling components during evolution. Recently, it was reported that a filamentous charophycean alga, *Klebsormidium flaccidum*, does not have auxin-mediated transcriptional regulation, suggesting its evolution around land adaptation of plants. Here, we revealed that a liverwort species, *Marchantia polymorpha*, has a simplified but complete auxin-mediated transcription mechanism compared with other land plant species. Despite the minimal auxin system, *M*. *polymorpha* exhibited pleiotropic auxin responses in morphogenesis including roles in organ differentiation and cell elongation. Phylogenetic and experimental analyses revealed that the three ARF transcription factors in *M*. *polymorpha* had different transcriptional activities and interacted in various combinations with different affinities. This suggests that the auxin-mediated transcriptional regulation was established in the common ancestor of land plants, and might be correlated with the change in body plans at this time in the evolution of land plants.

## Blurb

The liverwort *Marchantia polymorpha* has a minimal but required repertoire of components for auxin-mediated transcriptional regulation, which is sufficient for achieving the simple yet multi-dimensional body plan.

## Introduction

In angiosperms, the plant hormone auxin regulates many aspects of growth and development such as axis formation during embryogenesis [1], initiation of leaf primordia at the shoot apical meristem [2], root development [3], and tropic responses to light or gravity [4,5]. A major auxin-signaling pathway is transcriptional regulation mediated by a co-receptor consisting of TRANSPORT INHIBITOR RESPONSE1/AUXIN SIGNALING F-BOX (TIR1/AFB) and AUXIN/INDOLE-3-ACETIC ACID (AUX/IAA). In addition, AUXIN BINDING PROTEIN 1 (ABP1), which is evolutionarily conserved from charophyte green algae to seed plants, is known as an extracellular auxin receptor involved in rapid re-orientation of microtubules [6–9]. Studies in Arabidopsis and other angiosperms have revealed that auxin perception by the TIR1/AFB-AUX/IAA co-receptor triggers transcriptional regulation mediated by the AUXIN RESPONSE FACTOR (ARF) transcription factors, which directly bind to *cis*-elements (auxin responsive elements, or AuxREs) of auxin responsive genes and positively or negatively regulates the expression [10]. In the absence of auxin, AUX/IAAs bind to ARFs through their C-terminal regions, called domains III/IV, of the respective proteins and the complex represses the expression of auxin responsive genes [11,12]. Auxin functions as “molecular glue” that stabilizes the interaction between the F-box protein TIR1/AFB and the transcriptional repressor AUX/IAA [13,14]. This interaction promotes ubiquitination of AUX/IAA by the ubiquitin ligase complex that contains TIR1/AFB and subsequent degradation of AUX/IAA by the 26S proteasome [15], which liberates the ARFs and allows them to play their roles in transcriptional regulation.

In Arabidopsis, an ensemble of 29 AUX/IAAs and 23 ARFs is believed to regulate various auxin responses [16,17]. However, the high level of genetic redundancy of these transcription factors and the complex body plan composed of various organs make it difficult to depict a comprehensive picture of auxin regulatory events consisting of interactions and feedback among multiple factors.

Auxin responses are also observed in basal land plant lineages, such as the bryophytes (liverworts, mosses, and hornworts), and green algal lineages related to land plants, the charophytes [18]. Whole genome sequencing approaches of the moss *Physcomitrella patens* and the lycophyte *Selaginella moellendorffii*, a member of the basal lineage of vascular plants, have revealed that these two species have orthologues of basic components for auxin-mediated transcriptional regulation with relatively lower redundancy than observed in flowering plants [19,20]. Additionally, it was reported that *P*. *patens* possesses the auxin perception mechanism mediated by TIR1/AFB and AUX/IAA, which regulates the chloronema-caulonema transition and rhizoid formation [21]. On the other hand, there is limited knowledge about auxin-mediated regulatory systems in green algae. It is generally accepted that the ancestor of land plants was closely related to charophytes [22]. A recent study on the draft genome sequence of *Klebsormidium flaccidum*, a filamentous charophyte lacking differentiation of specialized cells, reported the absence of *TIR1*/*AFB*, *AUX*/*IAA*, and *ARF* genes in its genome [23], suggesting that auxin-mediated transcriptional regulation evolved subsequent to the divergence of *Klebsormidium* and the lineage leading to land plants.

*Marchantia polymorpha* is a liverwort species belonging to the earliest diverging clade of extant land plants [22] and has long history as an experimental organism. *M*. *polymorpha* is a complex thalloid liverwort and spends most of its life cycle as a haploid flat thallus which grows apically and has distinct dorsiventrality. On the dorsal side, air chambers are regularly arranged [24], and asexual reproductive organs, gemma cups and gemmae, are repeatedly formed [25]. On the ventral side of thallus, scales and rhizoids are produced [26,27]. *M*. *polymorpha* is dioecious and produces gametangiophores (archegoniophores that produce egg cells and antheridiophores that produce sperms) for sexual reproduction under certain environmental conditions [28,29]. Following fertilization, a diploid zygote develops into a multicellular sporophyte, or embryo, on archegoinophores, in which a set of cells undergo meiosis, resulting in a sporangium producing single-celled haploid spores [30].

The most common endogenous auxin, indole-3-acetic acid has been detected in *M*. *polymorpha* [31]. Application of exogenous auxin has revealed that auxin is involved in rhizoid initiation and elongation [32–34], thallus growth [34,35], regeneration from excised thalli [36], and apical dominance [37] in *M*. *polymorpha* and in dormancy of gemma in *Lunularia*, a related liverwort genus [38].

Recently, *M*. *polymorpha* has received attention for its critical evolutionary position [39]. Molecular genetic tools including transformation techniques [40,41], homologous recombination [42], and CRISPR/Cas9-mediated genome editing [43] have been developed. By applying molecular techniques, we demonstrated that the auxin response reporter which expresses β-glucuronidase under the soybean-derived GH3 promoter (_*pro*_ *GH3*:*GUS*) specifically responds to auxin in a dose-dependent manner [34], suggesting a possible conservation of regulatory machinery for auxin-mediated transcriptional activation in *M*. *polymorpha*.

In this study, we performed an in silico search for auxin signaling factors in *M*. *polymorpha*, and demonstrate that it has a minimal but complete auxin-mediated transcriptional system. We also describe critical roles of AUX/IAA-mediated auxin signaling in *M*. *polymorpha* development throughout its life cycle. Moreover, we investigated protein interactions and functional diversities of ARFs in *M*. *polymorpha*. From these results, we discuss how various auxin responses are regulated using the minimal set of auxin signaling factors in *M*. *polymorpha*.

## Results

### Identification of auxin signaling factors in *M*. *polymorpha*

To investigate whether *M*. *polymorpha* has basic components of auxin signaling, genes for known auxin signaling factors were surveyed by BLAST searches against *M*. *polymorpha* transcriptome and genome databases.

**Fig 1:**
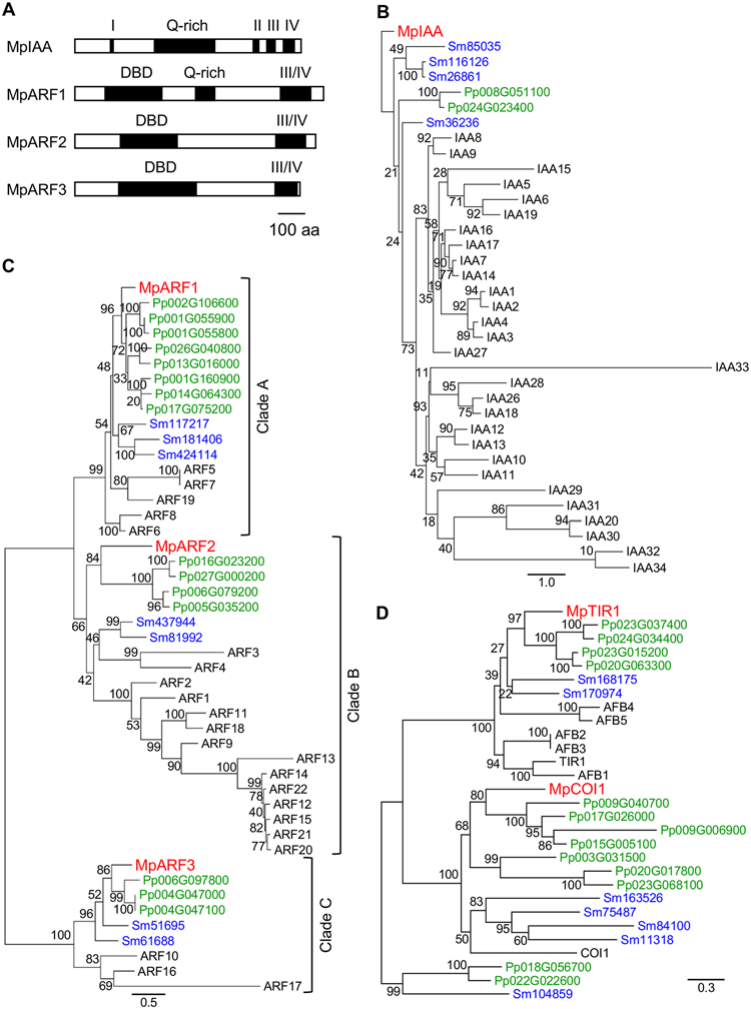
Auxin signaling factors in *M*. *polymorpha*. (A) Diagrams of domain structures of MpIAA and MpARFs. DBD: DNA-binding domain, Q-rich: glutamine-rich region, Roman numbers: domains I through IV. (B-D) Phylogenetic positions of MpIAA (B), MpARFs (C) and MpTIR1 (D) using the amino acid sequences from *M*. *polymorpha* (red), *P*. *patens* (green), *S*. *moellendorffii* (blue), and Arabidopsis (black). See S2 Table for sequence information. Numbers on the branches indicate bootstrap values. Bar in (A): 100 amino acid residues in length. Bars in (B-D): number of amino acid changes per branch length.

For *AUX/IAA*, only a single gene was found in the *M*. *polymorpha* genome (*MpIAA*), that contains four conserved domains (domains I to IV). In the N-terminus, the predicted MpIAA protein has a domain I motif (LxLxL), which is predicted to bind directly to the transcriptional co-repressor TOPLESS (TPL; Figs 1A and S1) [44]. In the C-terminus, MpIAA contains a domain II sequence, the AUX/IAA degron, and domains III and IV, which comprise a protein-protein interaction domain (Figs 1A and S1). In the domains III/IV of MpIAA, invariant lysine and acidic residues, which are shown to be important for AUX/IAA-ARF oligomerization in Arabidopsis, are conserved (S1 Fig) [45,46]. The amino acid sequence of MpIAA is considerably longer (825 amino acid residues) as compared to AUX/IAA proteins of vascular plants, and contains an additional glutamine-rich stretch between domains I and II. To understand if this structure is evolutionarily conserved, we obtained partial sequence of AUX/IAA homologues from two other Marchantiales species, *Conocephalum conicum* and *C*. *japonicum*, via degenerate RT-PCR. The *Conocephalum* sequences also contain long glutamine-rich regions in their N-termini, implying that this region has a conserved function among at least the Marchantiales (S1 Fig). In phylogenetic analyses, MpIAA resided in a clade with *P*. *patens* and *S*. *moellendorffii* distinct from the clade of Arabidopsis AUX/IAA sequences (Fig 1B).

The *M*. *polymorpha* genome encodes three ARFs (MpARF1, MpARF2, and MpARF3). The three MpARFs have a structure common to typical ARFs, containing a B3-like DNA binding domain (DBD) and a protein-protein interaction motif, called domains III and IV (Figs 1A and S2). Between the DBD and domain III, MpARF1 harbors a glutamine-rich region (Fig 1A), which is known as a feature of activator ARF members [10]. Finet *et al*. (2013) classified 224 ARF proteins across diverse land plants into three clades: clade A, clade B and clade C [47]. Phylogenetic analysis showed that MpARF1 belonged to the clade A, which represents activator ARF proteins such as ARF5/MONOPTEROS (MP) of Arabidopsis (Fig 1C). MpARF2 resided in clade B (Fig 1C). In Arabidopsis, some ARF members in this clade have been shown to function as transcriptional repressor [10,48]. MpARF3 has a relatively longer DBD, and its domains III and IV show lower similarity to ARFs in the clades A and B (Figs 1A, S2A, and S2B). *MpARF3* mRNA contains the possible target sequence of microRNA160 (S2C Fig). These features are common with Arabidopsis ARF10 and ARF16, which possibly function as repressors [49]. Phylogenetic analysis supported the placement of MpARF3 in the clade C including Arabidopsis ARF10, ARF16, and ARF17 (Fig 1C). In summary, MpARF1, MpARF2, and MpARF3 were phylogenetically classified into the clades A, B, and C, respectively, suggesting that three functionally diverged types of ARFs existed in the common ancestor of extant land plants.

BLAST searches revealed that *M*. *polymorpha* genome harbors two genes that, respectively, exhibit high similarity to TIR1/AFB and a jasmonic acid receptor, CORONATINE INSENSITIVE1 (COI1), of Arabidopsis. One encodes a protein with 54% identity to Arabidopsis TIR1 and is phylogenetically classified in the TIR1/AFB clade (named MpTIR1, Figs 1D and S3). The other sequence is 44% identical to COI1, and phylogenetic analysis supported that this sequence belonged to the COI1 clade (Fig 1D). These results suggest that *M*. *polymorpha* has only one TIR1/AFB auxin receptor.

Taken together, *M*. *polymorpha* has all required components for auxin-mediated transcriptional regulation with minimal genetic redundancy. We also performed BLAST and HMMER searches using another auxin receptor, ABP1, as query. ABP1 is broadly conserved in the green lineage including charophytes green algae [7]. However, to our surprise, no homologue of ABP1 was found in the *M*. *polymorpha* genome.

### Repression of auxin responses by domain II-modified MpIAA

**Fig 2:**
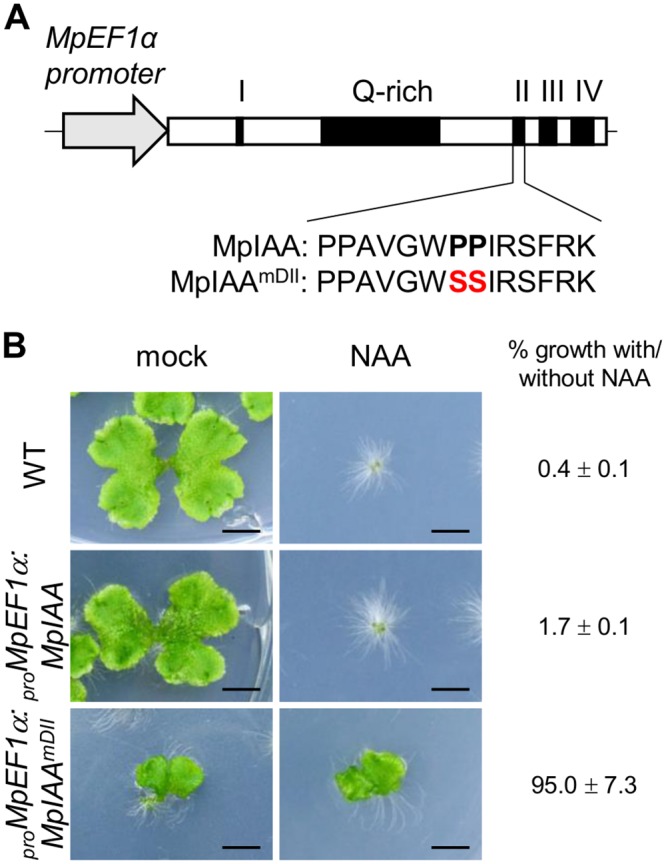
Effects of mutations in domain II of MpIAA on auxin sensitivity. (A) Diagram of _*pro*_ *MpEF1α*:*MpIAA* and _*pro*_ *MpEF1α*:*MpIAA* ^*mDII*^. Two conserved proline residues in domain II were substituted with serine. (B) Resistance to auxin by domain II-modified MpIAA expression. Photographs of WT, _*pro*_ *MpEF1α*:*MpIAA* and _*pro*_ *MpEF1α*:*MpIAA* ^*mDII*^ plants cultured with exogenous 3 μM NAA or under mock conditions for 2 weeks. Bars: 5 mm. The values indicate area ratios of 2-week-old thallus grown in the presence of NAA to that grown under mock conditions with SE (n = 12).

Studies in angiosperms, including Arabidopsis, have shown AUX/IAA as the key component in auxin perception and signaling. Dominant mutations in domain II of AUX/IAA inhibit its auxin-dependent degradation and results in auxin resistance [50]. Because *M*. *polymorpha* has only the single AUX/IAA, we focused on *MpIAA* to investigate the mechanism and function of auxin signaling in *M*. *polymorpha*. To examine if *MpIAA* is involved in auxin signaling in *M*. *polymorpha*, we generated transgenic plants which expressed MpIAA with or without substitutions of two conserved proline residues in the domain II degron sequence into serines under the control of the *MpELONGATION FACTOR 1α* constitutively active promoter (_*pro*_ *MpEF1α*:*MpIAA* ^*mDII*^ and _*pro*_ *MpEF1α*:*MpIAA*; Fig 2A) [51] and analyzed their responses to exogenous auxin. In the absence of auxin, _*pro*_ *MpEF1α*:*MpIAA* plants were indistinguishable from wild type (WT). In contrast, in the absence of exogneous auxin _*pro*_ *MpEF1α*:*MpIAA* ^*mDII*^ plants displayed morphological defects such as dwarfism and curling of the thallus. In the presence of high concentrations of exogenous naphthaleneacetic acid (NAA), a synthetic auxin, _*pro*_ *MpEF1α*:*MpIAA* plants exhibited growth arrest and produced many rhizoids as reported for WT previously [34,52–55], while _*pro*_ *MpEF1α*:*MpIAA* ^*mDII*^ plants were insensitive to NAA treatment (Fig 2B). Thus, we conclude that MpIAA conveys auxin signaling through domain II function in a similar manner as that demonstrated in angiosperms.

**Fig 3:**
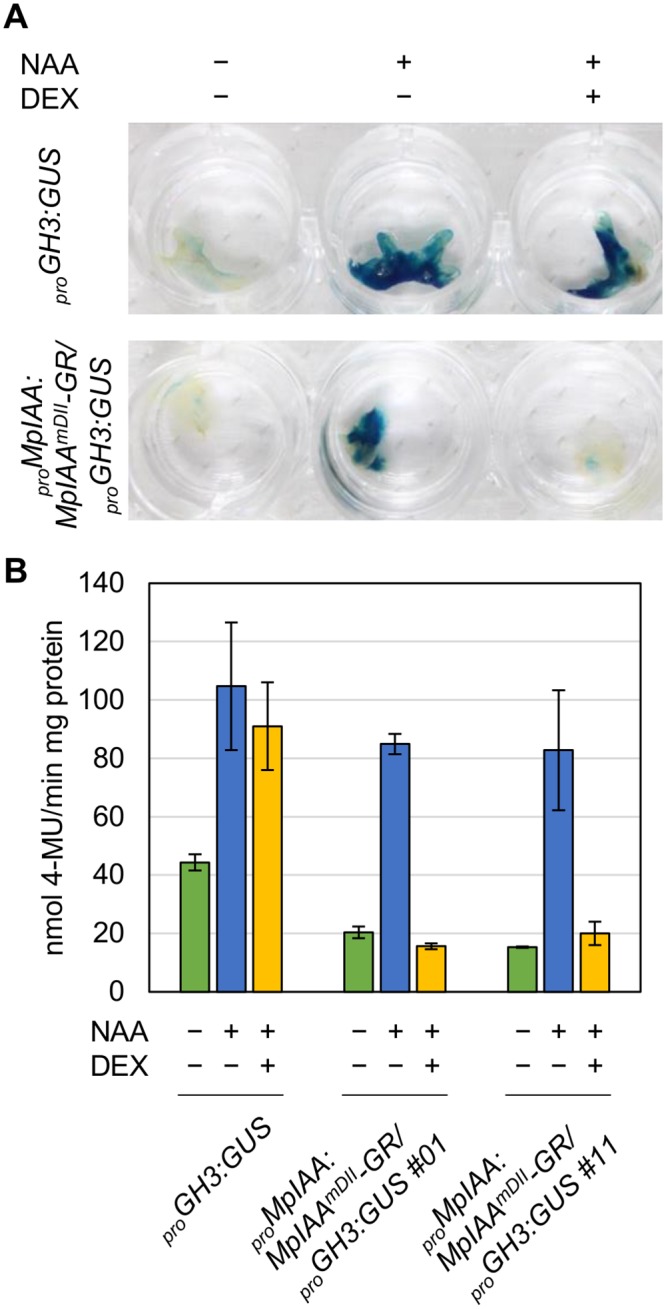
DEX-inducible system for repressing auxin responses. (A, B) GUS staining (A) and quantitative fluorometric assays (B) of _*pro*_ *GH3*:*GUS* and _*pro*_ *MpIAA*:*MpIAA* ^*mDII*^ *-GR/*_*pro*_ *GH3*:*GUS* transgenic plants. Each plant was treated with 10 μM NAA and/or 10 μM DEX for 12 h. Error bars: SE (n = 3).

We observed that the expression level of transgenes in the lines obtained was significantly lower in _*pro*_ *MpEF1α*:*MpIAA* ^*mDII*^ than that of _*pro*_ *MpEF1α*:*MpIAA* (S4 Fig), suggesting that high levels of expression of *MpIAA* ^*mDII*^ driven by the *MpEF1α* promoter might be deleterious. Therefore, we utilized an inducible system in which protein nuclear localization is modulated by a glucocorticoid receptor (GR) domain [56,57], to investigate in more detail the function of *MpIAA* and to determine developmental processes where auxin-mediated transcriptional regulation is involved in the life cycle of *M*. *polymorpha*. We generated transgenic plants expressing a chimeric protein of *MpIAA* ^*mDII*^ C-terminally fused with GR under the control of the *MpIAA* promoter (_*pro*_ *MpIAA*:*MpIAA* ^*mDII*^ *-GR*). In these transgenic plants auxin signaling should be repressed upon treatment with dexamethasone (DEX). To confirm that this experimental strategy works in plants, we first introduced _*pro*_ *MpIAA*:*MpIAA* ^*mDII*^ *-GR* into the *M*. *polymorpha* lines harboring the auxin response reporter _*pro*_ *GH3*:*GUS* [34]. Twelve hours of exogenous auxin treatment increased GUS activity in _*pro*_ *GH3*:*GUS* control plants. DEX treatment applied to _*pro*_ *MpIAA*:*MpIAA* ^*mDII*^ *-GR/*_*pro*_ *GH3*:*GUS* plants completely repressed auxin-dependent expression of the *GUS* reporter gene, whereas DEX treatment did not affect auxin-induced GUS activity in _*pro*_ *GH3*:*GUS* plants (Fig 3). These results suggest that the presumable accumulation of MpIAA^mDII^ protein in the nucleus represses an auxin-dependent transcriptional response as has been shown in Arabidopsis [58]. Taken together, the mechanism of AUX/IAA-mediated auxin response is conserved in *M*. *polymorpha*.

### Regulation of cell expansion by *MpIAA*-mediated auxin signaling

**Fig 4:**
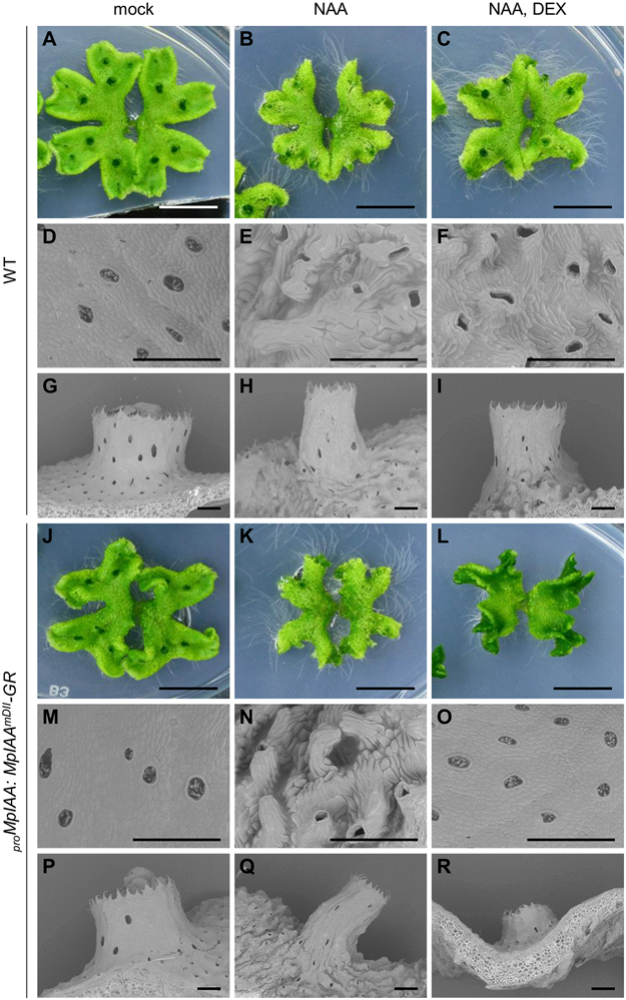
Effects of exogenous auxin on the morphology and cell shape. WT (A-I) and _*pro*_ *MpIAA*:*MpIAA* ^*mDII*^ *-GR* plants (J-R) were grown for 12 days in the absence of both NAA and DEX, then grown under mock condition (A, D, G, J, M, P), with 10 μM NAA (B, E, H, K, N, Q), or with 10 μM NAA and 10 μM DEX (C, F, I, L, O, R). At day 7 post treatment, photographs (A-C, J-L; bars: 10 mm) and scanning electron micrographs (D-I, M-R; bars: 500 μm) were taken.

We next examined the significance of *MpIAA*-mediated auxin signaling in the regulation of cellular morphology in *M*. *polymorpha*. We first analyzed the morphological and cellular responses of *M*. *polymorpha* thalli to exogenously supplied auxin. In the WT thallus, NAA treatment caused epinasty of thalli (Fig 4A and 4B), protrusion of air chambers (Fig 4D and 4E), and elongation of gemma cups (Fig 4G and 4H). Quantification of cell parameters revealed directional expansion of dorsal epidermal cells (S5 Fig), which therefore may be responsible for the above phenotypes. In the absence of DEX, _*pro*_ *MpIAA*:*MpIAA* ^*mDII*^ *-GR* plants responded to NAA as did WT (Fig 4J, 4K, 4M, 4N, 4P, and 4Q). Treatment of _*pro*_ *MpIAA*:*MpIAA* ^*mDII*^ *-GR* plants with DEX alleviated the NAA-induced phenotypes (Figs 4L, 4O, 4R, and S5), whereas the same treatment to WT thallus did not (Figs 4C, 4F, 4I, and S5). These results suggest that cell expansion in dorsal epidermal tissue is promoted by auxin through *MpIAA*-mediated transcriptional regulation.

### Expression specificity of *MpIAA* in the life cycle

**Fig 5:**
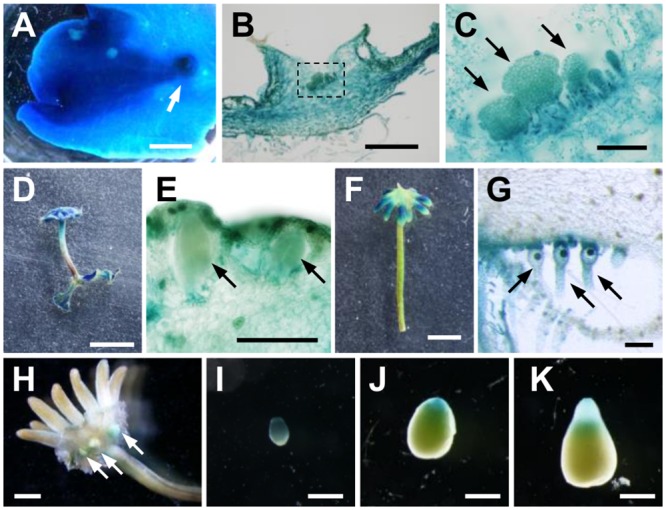
Expression pattern of *MpIAA* throughout the life cycle. GUS staining of _*pro*_ *MpIAA*:*GUS* plants. (A) 3-week-old thallus. Arrow: gemma cup. (B) Longitudinal section of gemma cup. (C) Magnified view of the region shown by dotted line in (B). Arrows: developing gemmae. (D, E) Overview (D) and longitudinal section (E) of antheridiophore. The arrows represent antheridia. (F, G) Overview (F) and longitudinal section (G) of archegoniophore. Arrows: archegonia. (H-K) Sporophytes generated by crossing WT female and _*pro*_ *MpIAA*:*GUS* male. (H) Overview of fertilized archegoniophore containing developing sporophytes (arrows). (I-K) Isolated developing sporophytes. The apices of the sporophytes are directed downward. Scale bars: 2 mm (A, H), 0.5 mm (B, I-K), 0.1 mm (C, E, G), 5 mm (D, F).

The results described thus far suggest the central role of the sole *AUX/IAA*, *MpIAA*, in the auxin-mediated transcriptional regulation in *M*. *polymorpha*. To investigate spatiotemporal expression pattern of *MpIAA*, we generated transgenic plants expressing *GUS* reporter gene under the regulation of *MpIAA* promoter (_*pro*_ *MpIAA*:*GUS*). High levels of GUS activity was observed throughout _*pro*_ *MpIAA*:*GUS* thalli including the gemma cups (Fig 5A). Cross-sections of the _*pro*_ *MpIAA*:*GUS* thallus revealed that GUS staining was observed in all layers of thallus tissue, including dorsal air chambers, gemma cups and developing gemmae, internal parenchymatous tissue, and ventral scales and rhizoids (Fig 5B and 5C).

We also observed expression during the reproductive phase. Antheridiophores showed strong GUS staining (Fig 5D), including GUS signal in antheridia and surrounding tissues (Fig 5E). Compared with antheridiophores, archegoniophores showed relatively weak GUS staining, which was observed in the tips of digitate rays (Fig 5F). Cross-sectional analysis revealed that intensive GUS activity was observed in archegonia including the egg cells (Fig 5G).

We also observed expression in the diploid sporophyte generation following fertilization of the haploid gametes. Strong GUS staining was observed in young sporophytes (Fig 5H and 5I), and during sporophyte development, a gradient of GUS activity was evident along apical-basal axis. GUS staining of apical sporogenous tissue was relatively weaker, and diminished as sporophyte matured, whereas that of the basal region consisting of the foot and seta remained (Fig 5I–5K). These GUS staining patterns in the sporophyte were observed in reciprocal crosses between _*pro*_ *MpIAA*:*GUS* and WT.

These results suggest that *MpIAA* is widely expressed in both gametophyte and sporophyte generations with some tissue specificity, and that *MpIAA*-mediated auxin signaling would function in various tissues and organs in both generations of the life cycle.

### Pleiotropic roles of *MpIAA*-dependent auxin signaling throughout life cycle

**Fig 6:**
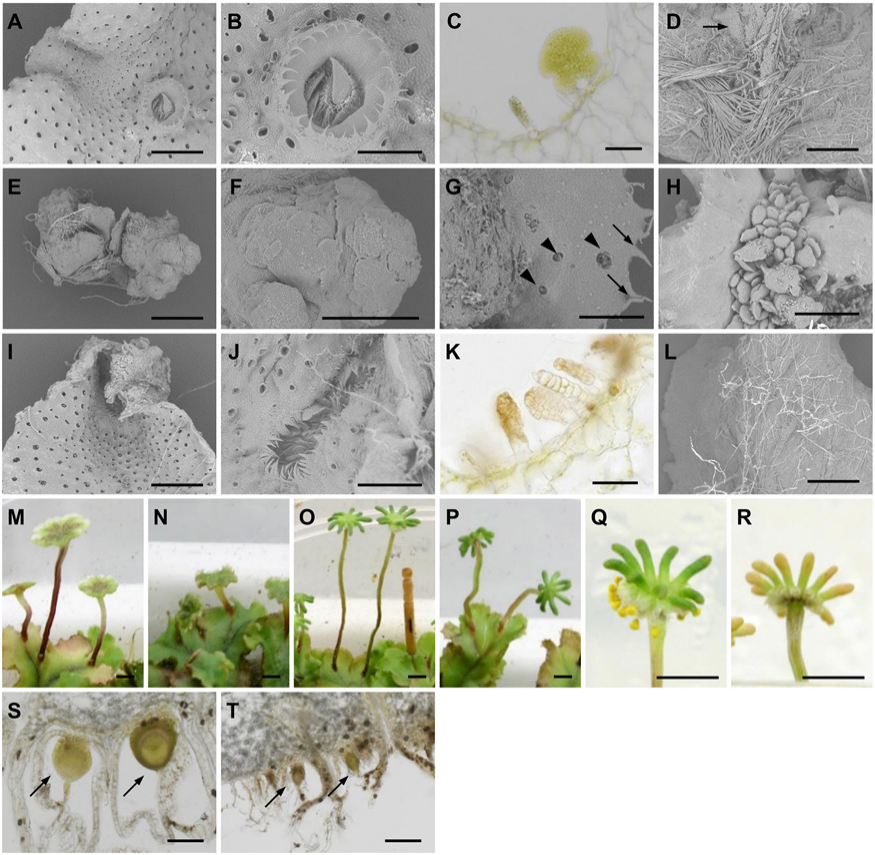
Morphological defects of _*pro*_ *MpIAA*:*MpIAA* ^*mDII*^ *-GR* plants. (A-D) _*pro*_ *MpIAA*:*MpIAA* ^*mDII*^ *-GR* plants grown for 14 days without DEX. (A) SEM of the dorsal side of the thallus. Air pores are visible as black dots. (B, C) SEM (B) and longitudinal section (C) of a gemma cup. (D) SEM of the ventral side of the thallus. Arrow: ventral scale. (E-H) _*pro*_ *MpIAA*:*MpIAA* ^*mDII*^ *-GR* gemmalings grown in the presence of 10 μM DEX for 14 days. SEM images are shown. Arrows: serrated structures reminiscent of gemma cup. Arrowheads: air pores. (I-L) _*pro*_ *MpIAA*:*MpIAA* ^*mDII*^ *-GR* plants grown in the absence of DEX for 7 days and then subsequently in the presence of 10 μM DEX for 7 days. (I, J) SEM images of the dorsal side of the thallus (I) and a gemma cup (J). (K) Longitudinal section of a gemma cup. (L) SEM image of the ventral side of the thallus. (M-P) Antheridiophores (M, N) and archegoniophores (O, P) of male and female plants, respectively, grown for 2 weeks under mock (M, O) or DEX-treated (N, P) conditions. (Q-T) Fertilized archegoniophores at 4 weeks (Q, R) or 2 weeks (S, T) after crossing without (Q, S) or with DEX treatment (R, T). DEX treatment was performed every one or two days, beginning the day after crossing. Arrows: developing sporophytes. Scale bars: 1 mm (A, D, E, I, L), 0.5 mm (B, F-H, J), 0.1 mm (C, K), 5 mm (M-R), 0.2 mm (S, T).

In _*pro*_ *MpIAA*:*MpIAA* ^*mDII*^ *-GR* plants, auxin responses could be repressed in a DEX-dependent manner (see above). In order to investigate tissue- or stage-specific functions of MpIAA-mediated auxin signaling, we analyzed morphological phenotypes of _*pro*_ *MpIAA*:*MpIAA* ^*mDII*^ *-GR* plants caused by repression of auxin responses during various developmental stages. Without DEX treatment, _*pro*_ *MpIAA*:*MpIAA* ^*mDII*^ *-GR* plants developed normal thalli with gemma cups and regularly-arranged air pores on their dorsal sides (Fig 6A). Gemma cups formed serrated structures on their rims and produced many gemmae from their bases (Fig 6B and 6C). On the ventral side of thallus, ventral scales and numerous rhizoids were observed (Fig 6D).

Compared to the control condition, gemmalings grown in the presence of 10 μM DEX for 14 days showed severe growth inhibition (Fig 6A and 6E). The eight gemmalings observed exhibited some or all of the following morphological abnormalities: five produced a cellular mass lacking dorsiventrality (Fig 6F), four formed air pores ectopically, and five produced serrated structures, which were reminiscent of the gemma cup rim (Fig 6G). Adventitious gemma-like multicellular bodies were frequently (six of the eight) formed as clusters on the surface of gemmalings (Fig 6H). We could not find any ventral scales in the apical regions of gemmalings treated with DEX. These results suggest critical roles of *MpIAA*-mediated auxin signaling in gemmaling growth and differentiation, especially with respect to ventral structures.

We then applied DEX treatment to 7-day-old thallus precultured in the absence of DEX. At this stage, gemmalings had developed into mature thalli with organogenesis in a proper dorsiventral topology. DEX treatment for 7 subsequent days conferred hyponasty resulting in V-shaped thalli (Fig 6I). Although gemma cups were observed on dorsal side of the DEX-treated thalli, the cups were shallow and elongated along with the apical-basal axis, generating many serrated structures (Fig 6J). At the bottom of gemma cups, in spite of the normal development of gemma primordia, mature gemmae did not develop (Fig 6K). On the ventral side, the number of rhizoids was decreased, especially smooth rhizoids (Fig 6L). These results suggest involvement of endogenous auxin and *MpIAA*-mediated transcriptional regulation in the harmonized growth of dorsal and ventral thallus tissues. *MpIAA*-mediated auxin response also functions in the development of gemma cups, gemmae and rhizoids.

To investigate the role of *MpIAA*-mediated auxin signaling in gametangiophore growth, we started periodical DEX treatment to gametangiophores after they became visible (smaller than 5 mm in height). DEX treatment resulted in short stalks, while, in the control condition, male and female gametangiophores had vertically elongated stalks (Fig 6M–6P). Additionally, DEX treatment to _*pro*_ *MpIAA*:*MpIAA* ^*mDII*^ *-GR* plants compromised the tropic growth of gametangiophore stalks (Figs 6M–6P and S6). These results suggest involvement of *MpIAA*-mediated auxin signaling in both tropic and differential growth, which was reported as responses in gametangiophores to exogenous auxin application [59].

Finally, we investigated *MpIAA*-mediated auxin signaling in sporophyte development. Without DEX treatment, sporophytes developed on archegoniophores, producing yellow sporangia in approximately 4 weeks after crossing (Fig 6Q and 6S). Periodical DEX treatment that was initiated on the day following crossing conferred developmental arrest of the sporophyte (Fig 6T). We did not observe any mature sporangia 4 weeks after crossing (Fig 6R). These results suggest that proper *MpIAA*-mediated auxin signaling is critical for sporophyte development.

### Protein-protein interaction between MpIAA and MpARFs

**Fig 7:**
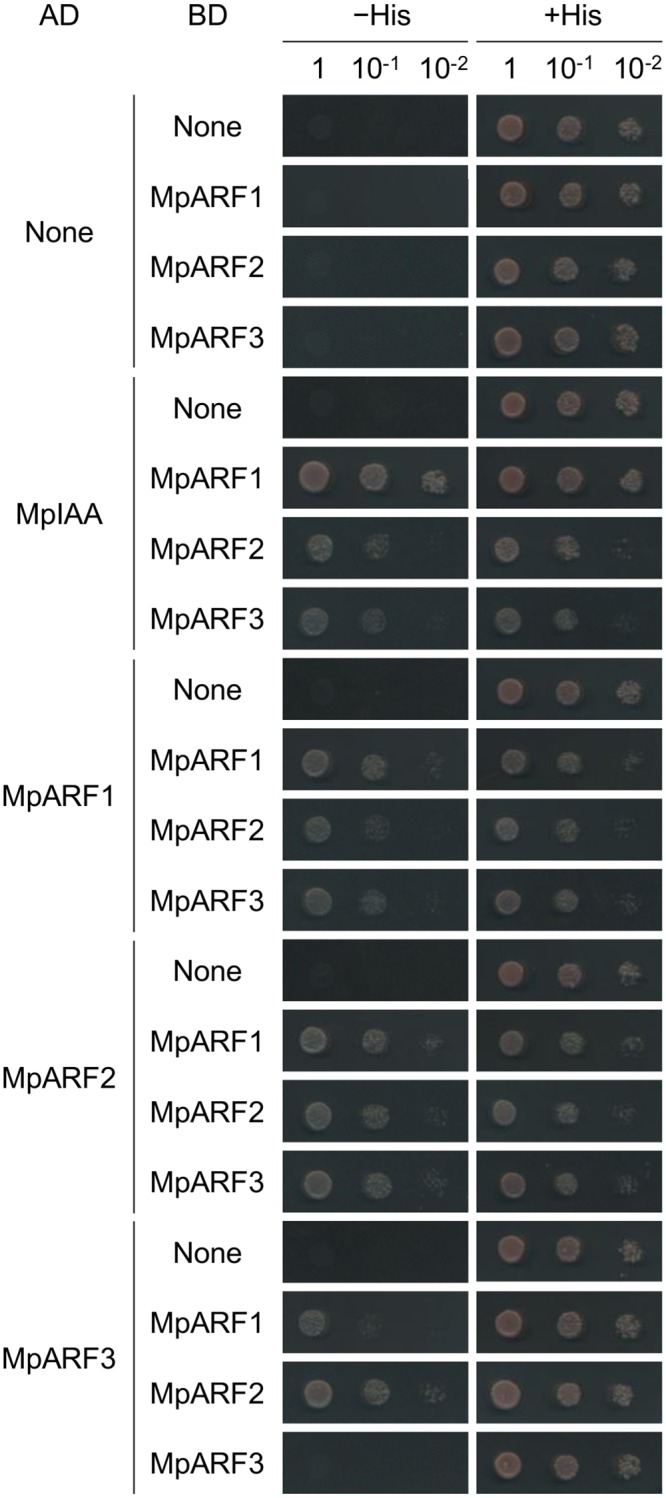
Protein-protein interactions between MpIAA and MpARFs in yeast. Yeast two-hybrid assays with the *HIS3* reporter. Ten-fold serial dilutions of overnight cultures were spotted on either nonselective +His (-Trp/-Leu) or selective-His (-His/-Trp/-Leu) media and grown for 2 days at 22°C. AD: proteins fused to VP16 activation domain. BD: proteins fused to lexA DNA-binding domain.

Phenotypic analysis of _*pro*_ *MpIAA*:*MpIAA* ^*mDII*^ *-GR* plants demonstrated that *MpIAA*-mediated auxin signaling regulates many aspects of growth and development of *M*. *polymorpha*. Therefore, our next question was how *M*. *polymorpha* generates various auxin responses using the simplified components for auxin-mediated transcriptional regulation. To tackle this question, we focused on the three *ARF* genes of *M*. *polymorpha*. We speculated that each MpARF might have a different specificity in protein-protein interactions and/or transcription activities. In Arabidopsis, interactions between AUX/IAA and ARF proteins have been examined by yeast two-hybrid (Y2H) assays and bimolecular fluorescence complementation (BiFC) [5,16]. To investigate if MpIAA and MpARFs have ability to form homo- or hetero-dimers, we first performed Y2H assays using C-terminal regions of MpIAA and MpARFs. Our Y2H assay showed that MpIAA could interact with all three MpARFs (Fig 7). Interactions between MpARFs were observed in all combinations except for MpARF3 homotypic interaction (Fig 7). We also examined the strengths of the observed Y2H interactions by quantitative measurements of 0β-galactosidase reporter activities (S1 Table). MpIAA showed high β-galactosidase activities with all MpARFs, but there were significant differences among the combinations. MpARF1 showed a notably higher activity in combination with MpARF1 than with the other MpARFs, while MpARF2 did with MpARF3, suggesting different affinities in the interactions among the three MpARFs.

**Fig 8:**
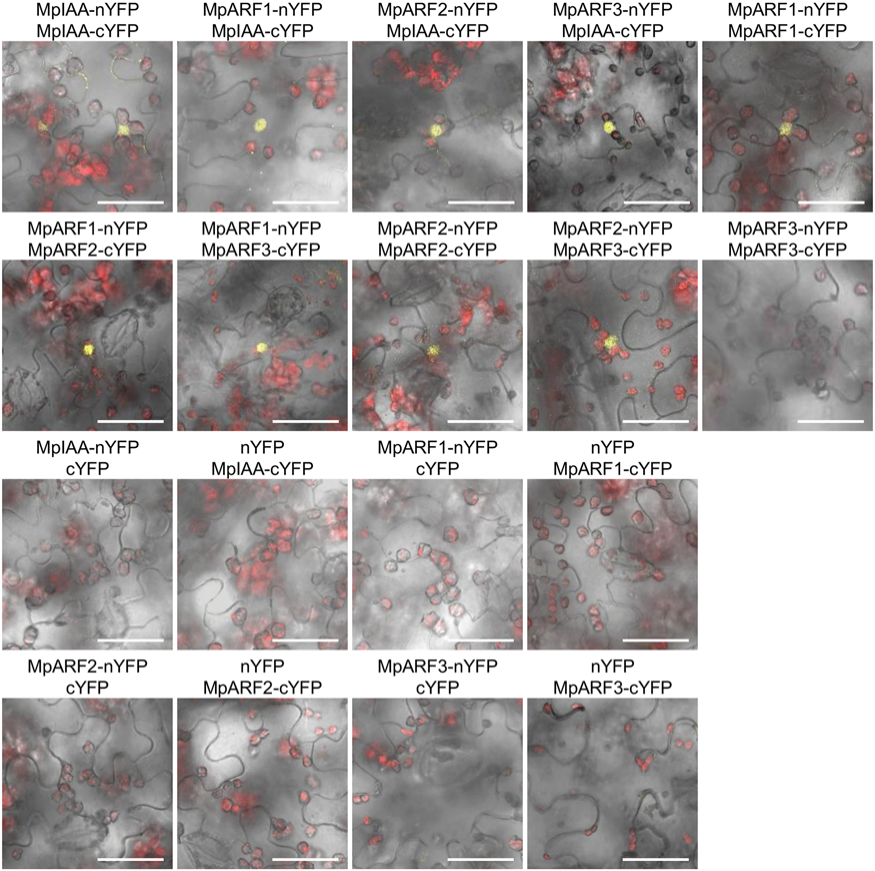
Protein-protein interactions between MpIAA and MpARFs *in planta*. BiFC assays of MpIAA and MpARF using *N*. *benthamiana* leaves. Confocal images of YFP (yellow) and chloroplast auto-fluorescence (red) were merged with bright-field images. Vectors containing only nYFP or cYFP was used as negative controls. Bars: 50 μm.

The interactions between MpIAA and MpARFs in planta were also examined by BiFC assay in *Nicotiana benthamiana* leaves. We analyzed all combinations of interactions between proteins fused to N-terminal and C-terminal halves of YFP under the condition where negative control experiments with empty vectors yielded no fluorescent signal. BiFC assays revealed that MpIAA interacted with MpIAA itself and all MpARFs. All combinations of MpARF-MpARF except for MpARF3-MpARF3 produced signal (Fig 8). These results confirmed protein interactions between MpIAA and MpARFs, as well as between MpARFs.

### Transcription activity of MpARFs

**Fig 9:**
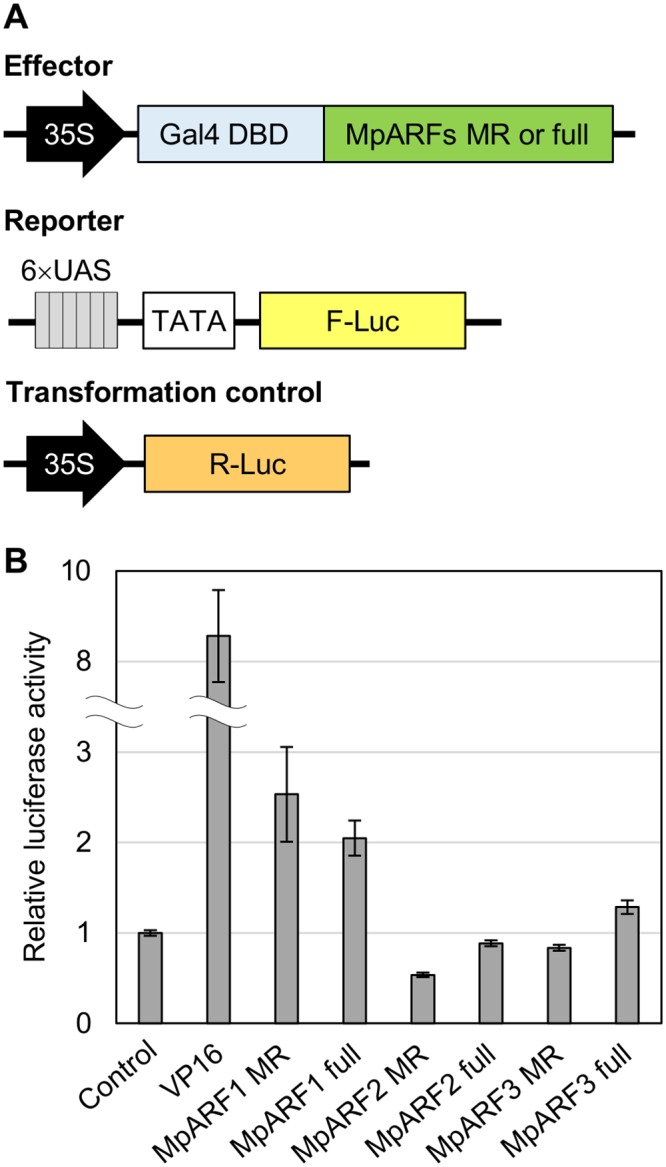
Tranactivation assay for MpARFs. (A) Diagrams of the constructs for dual luciferase assay. (B) Relative luciferase activity elicited by effector plasmid. The vector expressing only Gal4 DBD was used as a control. A virus-derived activation domain, VP16, was used for a positive control. See Material and Methods for effector vectors. Error bars: SE (n = 3).

To characterize the transcription activity of each MpARF, we performed transient transactivation assays using cultured tobacco BY-2 cells. The effector constructs carried full-length or the middle region sequences of MpARFs fused with the Gal4 DNA binding domain. The reporter vector expressed firefly luciferase (F-Luc) under the control of a promoter containing six repeats of the Gal4 binding site. As a transformation control, we prepared the plasmid carrying the *Renilla* luciferase (R-Luc) gene driven by the cauliflower mosaic virus 35S promoter (Fig 9A). These constructs were simultaneously introduced into BY-2 cells by particle bombardment. Transcriptional activity was evaluated by the relative activity of F-Luc to R-Luc. Both the middle region and full-length sequences of MpARF1 showed approximately two-fold higher activity than the effector expressing only Gal4 DBD. In the case of MpARF2, luciferase activity was lower than the control (Fig 9B). These results suggest that MpARF1 and MpARF2 can function as a transcriptional activator and repressor, respectively. We could characterize MpARF3 as neither an activator nor a repressor from this experiment, as the middle region and full-length sequences of MpARF3 showed just slightly lower and higher luciferase activities, respectively, than the control (Fig 9B). Taken together, our results suggest that *M*. *polymorpha* has three types of ARFs with different characteristics in their transcriptional activities.

## Discussion

### The origin and evolution of auxin-mediated transcriptional regulation in plants

Our results revealed that the liverwort *M*. *polymorpha* has a single *TIR1/AFB*, a single *AUX/IAA*, and three phylogenetically and functionally diverged *ARF* homologues. In Arabidopsis, it has been shown that AUX/IAA functions as a repressor through the interaction via domain I with the co-repressor TPL [44]. MpIAA has a conserved LxLxL motif in domain I (S1 Fig). The *M*. *polymorpha* genome encodes a homologue of TPL, and it is suggested that MpTPL is involved in auxin-mediated transcription (in an accompanying paper). In the present study, we showed that expression of domain II-modified MpIAA conferred an auxin-resistant phenotype and suppressed the transcriptional response to exogenously supplied auxin as monitored by _*pro*_ *GH3*:*GUS* (Figs 3 and 4). Additionally, it was reported that knock-down of *MpIAA* resulted in auxin hypersensitivity (in an accompanying paper). These results suggest that auxin-mediated degradation of MpIAA, presumably promoted by MpTIR1, is critical for transcriptional regulation. Our results also showed interaction between MpIAA and MpARFs through domains III/IV (Figs 7 and 8, S1 Table). Loss- and gain-of-function mutants of *MpARF1* show auxin-resistance and hypersensitivity, respectively (in an accompanying paper) [43]. Taken together, these results suggested that *M*. *polymorpha* possesses an auxin-mediated transcriptional regulation system that involves AUX/IAA and ARFs. Previous genomic analyses revealed that the lycophyte *S*. *moellendorffii* and the moss *P*. *patens* have all the basic components for auxin-mediated transcription [19,20], and that the filamentous charophyte alga *K*. *flaccidum* has none of the components [23]. Expressed sequences showing high similarity to the DBD and domains III/IV of ARFs were found in two other charophyte species, *Coleochaete orbicularis* and *Spirogyra pratensis*, respectively, that in some analyses represent the sister lineage to extant land plants [60,61]. Although it is still controversial whether aquatic ancestors of land plants had acquired the auxin-mediated transcriptional regulation, these data suggest that the origin of auxin responses using the three types of ARFs dates back to at least the last common ancestor of extant land plants.

In comparison with AUX/IAAs of vascular plants, the predicted amino-acid sequence of MpIAA, is much longer, and contains a long glutamine-rich region between domains I and II, which is conserved at least in the Marchantiales (Figs 1A and S1). Glutamine-rich domains are known to activate transcription in eukaryotes [62]. Activator ARFs, including MpARF1, also contain a glutamine-rich domain (Fig 1A) [10]. Since ARFs and AUX/IAAs also exhibit similarity in their C-terminal interaction domains, these genes likely evolved from a common ancestral gene. It is possible that the glutamine-rich domain of Marchantiales AUX/IAAs might be a remnant of the ancestral gene and may retain an unknown function in this lineage.

We previously demonstrated that _*pro*_ *GH3*:*GUS* activity could reflect the sites of endogenous auxin responses [34]. Expression of *MpIAA* was observed in various tissues including those showing high _*pro*_ *GH3*:*GUS* activities, such as the base of gemma cups, stalks and lobes of archegoniophores, antheridia and developing sporophytes (Fig 5). The significance of *MpIAA* expression in these tissues was supported by observation of phenotypes of _*pro*_ *MpIAA*:*MpIAA* ^*mDII*^ *-GR* plants (Fig 6J, 6K, and 6M–6T). These results suggest that auxin responses monitored by _*pro*_ *GH3*:*GUS* can be accounted for by MpIAA function. Interestingly, developmental defects of DEX-treated _*pro*_ *MpIAA*:*MpIAA* ^*mDII*^ *-GR* plants were also observed where no _*pro*_ *GH3*:*GUS* activity was detected, such as gemmalings and rhizoids (Fig 6E–6I and 6L). This could be possibly due to a high threshold of _*pro*_ *GH3*:*GUS* expression in response to auxin, or limitation of this reporter only representing the transcriptional activity mediated by activator-type ARF (MpARF1). If the latter is the case, non-activator-type ARFs (MpARF2 and MpARF3) could have important developmental roles in *M*. *polymorpha*.

### Evolutionarily conserved roles of auxin-mediated transcription in land plants

Auxin regulates many aspects of plant growth and development via modulating cell differentiation and expansion. In Arabidopsis, *ARF10* belonging to the same clade as *MpARF3* regulates cell totipotency in cultured cells [63]. In the moss *P*. *patens*, gain-of-function *AUX/IAA* mutants showed delayed caulonema differentiation from chloronema [21]. The present study showed that gemmaling produced undifferentiated cell mass by repression of *MpIAA*-mediated auxin signaling (Fig 6), suggesting that auxin-mediated transcription regulates cell differentiation in *M*. *polymorpha*. Consistently, transgenic plants expressing a bacterial auxin inactivating enzyme produced undifferentiated cell mass (in an accompanying paper). With respect to cell elongation, various mutants of *AUX/IAA*, *ARF*, and *TIR1/AFB* genes in Arabidopsis show defects in cell expansion and tropic responses [4,5,64–67]. Our study revealed that *MpIAA*-dependent auxin signaling regulated directional elongation of epidermal cells and tropic responses (Figs 4, 6M–6P, S5, and S6). These results suggest that regulation of cell differentiation and expansion by auxin-mediated transcription was already present in the common ancestor of land plants.

In Arabidopsis, it is reported that early-phase auxin-induced hypocotyl elongation occurs independently of TIR1/AFB-mediated transcription [68]. ABP1 has been proposed to be another auxin receptor that rapidly activates cell expansion in transcription-independent manner in angiosperms [6,69–72]. ABP1 is also found in green algae, although its function in these taxa remains to be elucidated [7,23,60]. To our surprise, no homologue of ABP1 was found in the *M*. *polymorpha* genome, suggesting that *M*. *polymorpha* lost ABP1 during evolution. This brings up new open questions, such as when was ABP1 function in auxin-mediated cell elongation established and what is the ancestral role of ABP1 in the plant and land plant lineages?

One of the important roles of auxin in plant development is axis formation. The present study revealed that the expression pattern of *MpIAA* exhibited a gradient along the apical-basal axis in the sporophyte, similar to the auxin-response reporter _*pro*_ *GH3*:*GUS* (Fig 5I–5K). Repression of *MpIAA*-mediated auxin signaling caused arrest of sporophyte development (Fig 6Q–6T). In the moss *P*. *patens*, it has been reported that the expression pattern of _*pro*_ *GH3*:*GUS* changes dynamically along apical-basal axis during sporophyte development, and that defects in auxin transport causes abnormal morphology of sporophyte [73,74]. In Arabidopsis, *IAA12/BODENLOS* and *ARF5/MP* are involved in formation of apical-basal axis in embryogenesis [75,76]. These results suggest that land plants would have common auxin-mediated mechanism for apical-basal axis formation during embryogenesis after fertilization.

Past studies showed that in *M*. *polymorpha* excessive exogenous auxin treatment promoted of rhizoid formation both dorsal and ventral sides of gemmalings, and thus proposed involvement of auxin into dorsiventral patterning of thallus [32,34,35,55]. The present study revealed that repression of *MpIAA*-mediated auxin signaling inhibited development of rhizoids and ventral scales (Fig 6). These results at least suggest *MpIAA*-mediated auxin signaling promotes the development of ventral tissues. It would be intriguing to clarify whether auxin mediates it directly or through dorsiventral axis formation.

### Diverse auxin responses by minimum components

In Arabidopsis, it is thought that the complex transcriptional regulation using 29 AUX/IAAs and 23 ARFs underlies robust auxin responses [16]. In addition, various combinations of TIR1 and AUX/IAA proteins form co-receptor complex with a wide range of auxin-binding affinities and show various auxin sensitivity of AUX/IAA degradation, which also contributes to complex auxin responses in angiosperms [77,78]. In this study, we demonstrated that the liverwort *M*. *polymorpha* regulates various developmental processes with a minimized auxin-mediated transcription system. Because *M*. *polymorpha* has only one *AUX/IAA* and one *TIR1/AFB* orthologue (Fig 1), it was expected that the variation in auxin responses could be attributed to the functional diversification of the three MpARFs. Our results showed that the three ARFs in *M*. *polymorpha* are phylogenetically diverged and have different transcriptional activities (Figs 1C and 9B). Furthermore, different binding affinities of MpIAA observed with the three MpARFs (S1 Table) suggest different auxin responsiveness, and MpARF-MpARF interactions (Figs 7 and 8) could add a higher level of regulation. This is also supported by the analyses of chimeric protein of TPL fused with domains III/IV of MpIAA and MpARFs (in an accompanying paper). Recently, crystal structure analyses revealed that AUX/IAA and ARF proteins multimerize through domain III/IV, and that the DBDs of ARFs dimerize and function as a “molecular caliper” when they bind to palindromically orientated AuxREs [45,46,79]. Although the physiological significance of interactions between MpARFs thorough domains III/IV and potentially between DBDs is unclear, it is possible that *M*. *polymorpha* can regulate diverse auxin responses via a combination of protein interactions of functionally diverged ARFs.

Still, would it be possible to explain such various auxin-response outputs only by the protein interaction variations? Recent work shows that functionally diverged ARF proteins from Arabidopsis have little differences in their DNA-binding specificity [79], raising the idea that the variation of ARFs may not contribute much to target specificities. Given the single-copy existence of the activator ARF in *M*. *polymorpha*, it might be more plausible that outputs are pre-determined depending on the respective cell types and that auxin just modulates switches via MpARF1. This idea is consistent with the recently proposed model, where auxin is viewed as a signal that provides “impetus” to processes [80]. It is expected that investigation on how three MpARFs regulate pleiotropic auxin responses will provide insights into the mechanisms of eliciting the variety of auxin responses observed in land plants.

### Perspectives

The present study demonstrated that *M*. *polymorpha* has minimal but complete, relative to that known in flowering plants, auxin-mediated transcription system, regulating diverse morphological events including both cell expansion and differentiation. In addition to transcriptional regulation, various auxin responses can be generated by regulating auxin biosynthesis, metabolism, and transport. It is also necessary to investigate the regulation of these factors in *M*. *polymorpha*. We propose that *M*. *polymorpha* with a low genetic redundancy is a good model for investigating the evolution and mechanisms of morphogenesis controlled by auxin.

## Materials and Methods

### Plant material and growth condition

Male and female accessions of *M*. *polymorpha*, Takaragaike-1 (Tak-1) and Tak-2, respectively [28], were maintained asexually. F1 spores generated by crossing Tak-1 to Tak-2 or _*pro*_ *GH3*:*GUS* #21 [34], were used for transformation.

Gametangiophore formation was induced by far-red irradiation as described previously [28]. *M*. *polymorpha* was cultured on half strength Gamborg’s B5 medium [81] containing 1% agar under 50–60 μmol photons m^-2^ s^-1^ continuous white fluorescent light at 22°C unless otherwise defined.

### Gene identification and phylogenetic analysis

A similarity search for *M*. *polymorpha* genes was performed using BLAST against transcriptome and genome databases from on-going project by US Department of Energy Joint Genome Institute (http://www.jgi.doe.gov/). The transcriptome data contained 3.0 × 10^6^ reads by Roche 454 GS FLX and >10^10^ reads by Illumina Hi-Seq from >18 conditions/tissues in different growth stages. The genomic DNA was sequenced under the coverage of 26.7× and 54.0× by Roche 454 GS FLX and Illumina Hi-Seq, respectively. The protein sequences of MpIAA, MpARFs and MpTIR1 were aligned with sequences listed in S2 Table. Partial cDNA sequences of *AUX/IAAs* in *C*. *conicum* and *C*. *japonicum* were amplified by degenerate RT-PCR using the primer set, degenerate-IAA_L2 and degenerate-IAA_R1. Primers used in this study are listed in S3 Table. PCR fragments were subcloned into pBC-SK+ and sequenced. These sequences were aligned using the MUSCLE program [82] implemented in Geneious software version 6.1.6 (Biomatters; http://www.geneious.com/) with default parameters. For phylogenetic analysis we used the C-terminal region (domain II-stop) of AUX/IAA, the DNA-binding domain of ARFs, and full length sequences of TIR1/AFBs. Phylogenic trees were generated by PhyML program version 2.1.0 [83] implemented in the Geneious software using the LG model and four categories of rate substitution. Tree topology, branch length, and substitution rates were optimized, and the tree topology was searched using the nearest neighbor interchange method. Bootstrap values were computed from 1000 trials. HMMER search for ABP1 homologues was performed against a database of six-frame translation products derived from the *M*. *polymorpha* transcript database, using the hmmscan program in HMMER3.1 (http://hmmer.org) with the raw HMM file for ABP1 downloaded from the Pfam database (http://pfam.xfam.org; ID: PF02041).

### Construction of plasmids for plant transformation

The coding sequence of *MpIAA* was amplified by RT-PCR using the primer set MpIAA_entry and MpIAA_stop, and cloned into pENTR/D-TOPO vector using the Gateway TOPO cloning kit (Life Technologies). Mutations in domain II were introduced by PCR using the primer set mDII_L3 and mDII_R3. Then the *MpIAA* and *MpIAA* ^*mDII*^ cassettes were transferred into pKIGWB2 using LR Clonase II (Life Technologies) according to the manufacture’s protocol, which generated _*pro*_ *MpEF1α*:*MpIAA* and _*pro*_ *MpEF1α*:*MpIAA* ^*mDII*^ constructs, respectively.

To generate a construct for _*pro*_ *MpIAA*:*GUS*, the genomic fragment covering from 5.2 kb upstream of putative start codon to the 52nd codon was amplified using the primer set MpIAA_usEntry and MpIAA_R9, and cloned into pENTR/D-TOPO vector. The resultant genomic fragment was transferred into pGWB3 [84] by LR Clonase II and translationally fused with *GUS* reporter gene.

To generate a construct for _*pro*_ *MpIAA*:*MpIAA* ^*mDII*^ *-GR*, the genomic fragment covering 5.2 kb upstream region and the coding sequence of *MpIAA* was amplified using the primer set MpIAA_usEntry and MpIAA_nonstop, and cloned into pENTR/D-TOPO vector. Mutations in domain II were introduced as described above. The glucocorticoid receptor hormone binding domain (GR) was amplified from pOpOn2.1 [85], and cloned into the *Asc*I site of pENTR/D-TOPO vector. The resultant cassette containing genomic fragment of *MpIAA* fused with *GR* was transferred into pGWB1 [84] or pMpGWB201.

### Transformation of *M*. *polymorpha*

Transformation of *M*. *polymorpha* was performed as described previously [40]. Independent T1 lines were isolated, and single G1 lines from independent T1 lines were established by subcultivating single gemmae which arose asexually from single initial cells [25,86]. Plants grown from gemmae of G1 lines (termed the G2 generation) were used for experiments.

### Histochemical assay for GUS activity

Histochemical assays for GUS activity were performed as described previously [34]. GUS stained gemma cups and gametangiophores were embedded in 6% agar block and sectioned into ~100-μm-thick slices with LinearSlicer PRO 7 (DOSAKA EM, Kyoto, Japan).

### Quantitative measurement of GUS activity

NAA and DEX treatments were performed by submerging plants into half-strength Gamborg’s B5 liquid medium [81] containing 10 μM NAA and/or 10 μM DEX for 12 h. GUS activity was then measured by monitoring cleavage of the β-glucuronidase substrate 4-methylumbelliferyl β-D-glucuronide (MUG) as described previously [29] with some modifications. After adjusting the concentration of extracted protein to 10 μg/70 μl extraction buffer, 20 μl of methanol and 10 μl of 10 mM MUG were added. After incubation at 37°C for 30 min, 900 μl of 200 mM sodium carbonate was added to stop the reaction. Fluorescence (460 nm emission/360 nm excitation) of liberated 4-methylumbelliferone (MU) was measured on Powerscan4 (DS Pharma Biomedical, Osaka, Japan).

### Phenotypic analysis of _*pro*_ *MpIAA*:*MpIAA* ^*mDII*^ *-GR* plants

In the vegetative phase, NAA and DEX treatments of _*pro*_ *MpIAA*:*MpIAA* ^*mDII*^ *-GR* plants were performed by growing on half-strength Gamborg’s B5 medium [81] containing 10 μM NAA and/or 10 μM DEX. In the reproductive phase, DEX treatment was performed by spraying 10 μM DEX solution every 1 or 2 days. For scanning electron microscopy, plant samples were frozen in liquid nitrogen and directly observed on a Miniscope TM3000 (HITACHI, Japan). Measurement of size and aspect ratio of epidermal cells, and curvature of gametangiophores were performed using ImageJ (http://imagej.nih.gov/ij/) from SEM and photographic images, respectively.

### Protein interaction analyses

For Y2H analyses the C-terminal regions of MpIAA (aa 627–825), MpARF1 (aa 783–928), MpARF2 (aa 751–879) and MpARF3 (aa 730–821) were cloned into pBTM116SBEN and pVP16PS, which were modified from pBTM116 and pVP16PS [87], using *Eco*RI/*Bam*HI and *Bam*HI/*Not*I site, respectively. Resultant constructs were transformed into the L40 *Saccharomyces cerevisiae* reporter strain [87], and transformants were selected on SD medium lacking tryptophan and leucine. Protein interactions were checked by histidine requirement or ONPG assays in the conventional method [87].

For BiFC analyses, C-terminal regions of MpIAA and MpARFs described above were cloned into pENTR/D-TOPO. Each insert fragment was introduced into pB4CY2 and pB4NY2 by the LR reaction. pB4CY2 and pB4NY2 were obtained from S. Mano, National Institute of Basic Biology, Japan. Preparation and infiltration of *Agrobacterium* cultures were performed as described previously [88]. Fluorescence from YFP (observation, 520 to 560 nm; excitation, 515 nm) was observed 20 to 21 h after infiltration. Fluorescent signals, chloroplast autofluorescence and bright-field images were captured using a confocal laser scanning microscope, FluoView 1000 (Olympus).

### Transient transactivation assay

cDNAs encoding full-length or middle region (MpARF1: aa 347–822, MpARF2: aa 388–764, MpARF3: aa 448–628) sequences of MpARF proteins were amplified with specific primers listed in S3 Table, and cloned into the vector to express them as a fusion protein with Gal4-DBD driven by the cauliflower mosaic virus 35S promoter [89] using the In-Fusion HD Cloning Kit (Clontech). A reporter plasmid containing six repeats of the Gal4 binding site and F-Luc, and the transformation control plasmid carrying R-Luc driven by the 35S promoter were described previously [89]. These constructs were introduced simultaneously into cultured tobacco BY-2 cells by bombardment using Biolistic PDS-1000/He Particle Delivery System (BIO-RAD). After 48 h incubation, F-luc and R-luc activities were assayed using the Dual-Luciferase Reporter Assay System (Promega) in accordance with the manufacture’s protocol. Luminescence was detected by Centro XS^3^ LB 960 Microplate Luminometer (Berthold). Luciferase activity was normalized by protein concentration.

### Semi-quantitative RT-PCR

Total RNA was extracted from 2-week-old thalli using TRIZOL Reagent (Life Technologies). First-strand cDNA was synthesized from 0.5 μg of total RNA with ReverTra Ace reverse transcriptase (Toyobo) and oligo(dT) primer. PCR amplification of the transgene-specific sequence was performed using the primer set MpIAA_dN2 and attB2_R. PCR amplification of the cDNA encoding the *EF1α* was performed as described before [90], and served as a control. These reactions were performed using the C1000 Thermal Cycler (Bio-Rad).

### Accession numbers

The genomic sequences from this article are available in DDBJ under the following accession numbers: AB981316 (*MpIAA*), AB981317 (*MpARF1*), AB981318 (*MpARF2*), AB981319 (*MpARF3*), AB981320 (*MpTIR1*), and AB981321 (*MpCOI1*).

## Supporting Information

S1 Fig Multiple alignment of AUX/IAA family. Protein sequences of AUX/IAA in *M*. *polymorpha*, *C*. *conicum*, *C*. *japonicum*, *P*. *patens*, *S*. *moellendorffii* and Arabidopsis were aligned using the MUSCLE program. Note that only partial sequences are shown for *C*. *conicum* and *C*. *japonicum*. Color boxes indicate domains I to IV. Blue and red asterisks indicate conserved basic or acidic residues, respectively.
(TIF)

S2 Fig Sequence analysis of MpARFs. (A, B) Multiple alignment of the DNA-binding domain (A) and C-terminal region (B) of ARF proteins. Protein sequences of ARFs in *M*. *polymorpha* and Arabidopsis were aligned using the MUSCLE program. Blue and red asterisks indicate conserved basic and acidic residues, respectively. (C) A possible target site of miR160 in *MpARF3* is shown with *ARF10*, *ARF16*, *ARF17* and *miR160a* of Arabidopsis. G-C and A-U base pairs are shown as colons, and a G-U base pair is shown as a dot.
(TIF)

S3 Fig Multiple alignment of TIR1/AFB family. Protein sequences of TIR1/AFBs in *M*. *polymorpha*, *P*. *patens*, *S*. *moellendorffii* and Arabidopsis were aligned using the MUSCLE program.
(TIF)

S4 Fig Expression analysis of transgenes by semi-quantitative RT-PCR. Expression levels of the transgenes in _*pro*_ *MpEF1α*:*MpIAA* and _*pro*_ *MpEF1α*:*MpIAA* ^*mDII*^ plants were analyzed by semi-quantitative RT-PCR using the introduced *MpIAA*-specific primers. PCR amplification of the cDNA encoding the *EF1α* was performed and served as a control. WT: wild type.
(TIF)

S5 Fig Quantitative analysis of the effects of exogenous auxin on cell morphology. WT and _*pro*_ *MpIAA*:*MpIAA* ^*mDII*^ *-GR* plants were grown for 12 days in absence of NAA and DEX, then subsequently grown either under the mock condition, with 10 μM NAA, or with 10 μM NAA and 10 μM DEX for 7 days. (A) SEM images of gemma cup. Bars: 0.5 mm. (B-C) The area (B) and the aspect ratio (C) of epidermal cells were measured by imageJ from the SEM images shown in (A). White broken lines indicate measured region. Error bars: SD (n = 50).
(TIF)

S6 Fig Tropic responses of _*pro*_ *MpIAA*:*MpIAA* ^*mDII*^ *-GR* gametangiophore. (A-H) Male (A-D) and female (E-H) thalli with visible gametangiophores of _*pro*_ *MpIAA*:*MpIAA* ^*mDII*^ *-GR* plants were transplanted to new pots (jiffy-7). After 3 days cultivation, water without (A, B, E, F) or with 10 μM DEX (C, D, G, H) was sprayed on the plants. After 3 h, the pots were orthogonally rotated. The images of day 0 (A, C, E, G) and day 1 (B, D, F, H) after rotation were taken. Bars: 5 mm. (I-J) Curvature of gametangiophores was determined as angle between direction of the stalk at day 0 and that at day 1, as shown in (J). Error bars: SD (n≥8). *: P<0.01. g: gravity.
(TIF)

S1 Table Interaction between MpIAA and MpARFs in Y2H system. (XLSX)

S2 Table List of sequences used for phylogenetic analysis. (XLSX)

S3 Table List of primers used in the present study. (XLSX)
