# Correction: Auxin-Mediated Transcriptional System with a Minimal Set of Components Is Critical for Morphogenesis through the Life Cycle in *Marchantia polymorpha*

**DOI:** 10.1371/journal.pgen.1005365

**Published:** 2015-06-26

**Authors:** Hirotaka Kato, Kimitsune Ishizaki, Masaru Kouno, Makoto Shirakawa, John L. Bowman, Ryuichi Nishihama, Takayuki Kohchi

A reference is omitted from the article. All sentences in the Discussion section which mention “in an accompanying paper” should cite Flores-Sandoval et al., 2015.

The reference is: Flores-Sandoval E, Eklund DM, Bowman JL (2015) A Simple Auxin Transcriptional Response System Regulates Multiple Morphogenetic Processes in the Liverwort *Marchantia polymorpha*. PLoS Genet 11(5): e1005207. doi:10.1371/journal.pgen.1005207.
