# Supplementary figures and images for: Auxin-Mediated Transcriptional System with a Minimal Set of Components Is Critical for Morphogenesis through the Life Cycle in Marchantia polymorpha
Source: PLoS Genet. 2015 May 28;11(5):e1005084. doi: 10.1371/journal.pgen.1005084 (PMC4447296; doi:10.1371/journal.pgen.1005084)

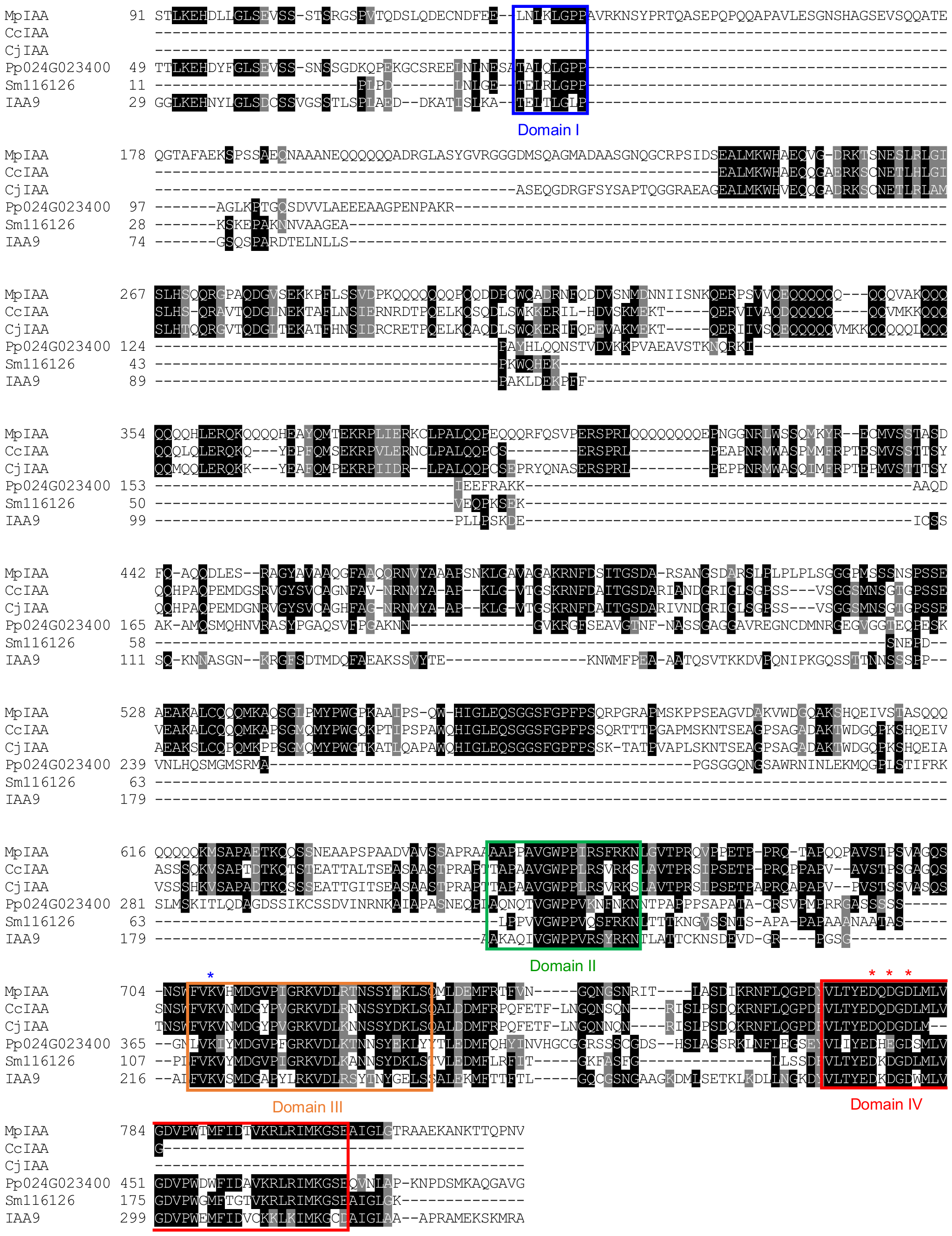

Supplement: S1 Fig — Protein sequences of AUX/IAA in M. polymorpha, C. conicum, C. japonicum, P. patens, S. moellendorffii and Arabidopsis were aligned using the MUSCLE program. Note that only partial sequences are shown for C. conicum and C. japonicum. Color boxes indicate domains I to IV. Blue and red asterisks indicate conserved basic or acidic residues, respectively. (TIF) [file pgen.1005084.s001.tif]

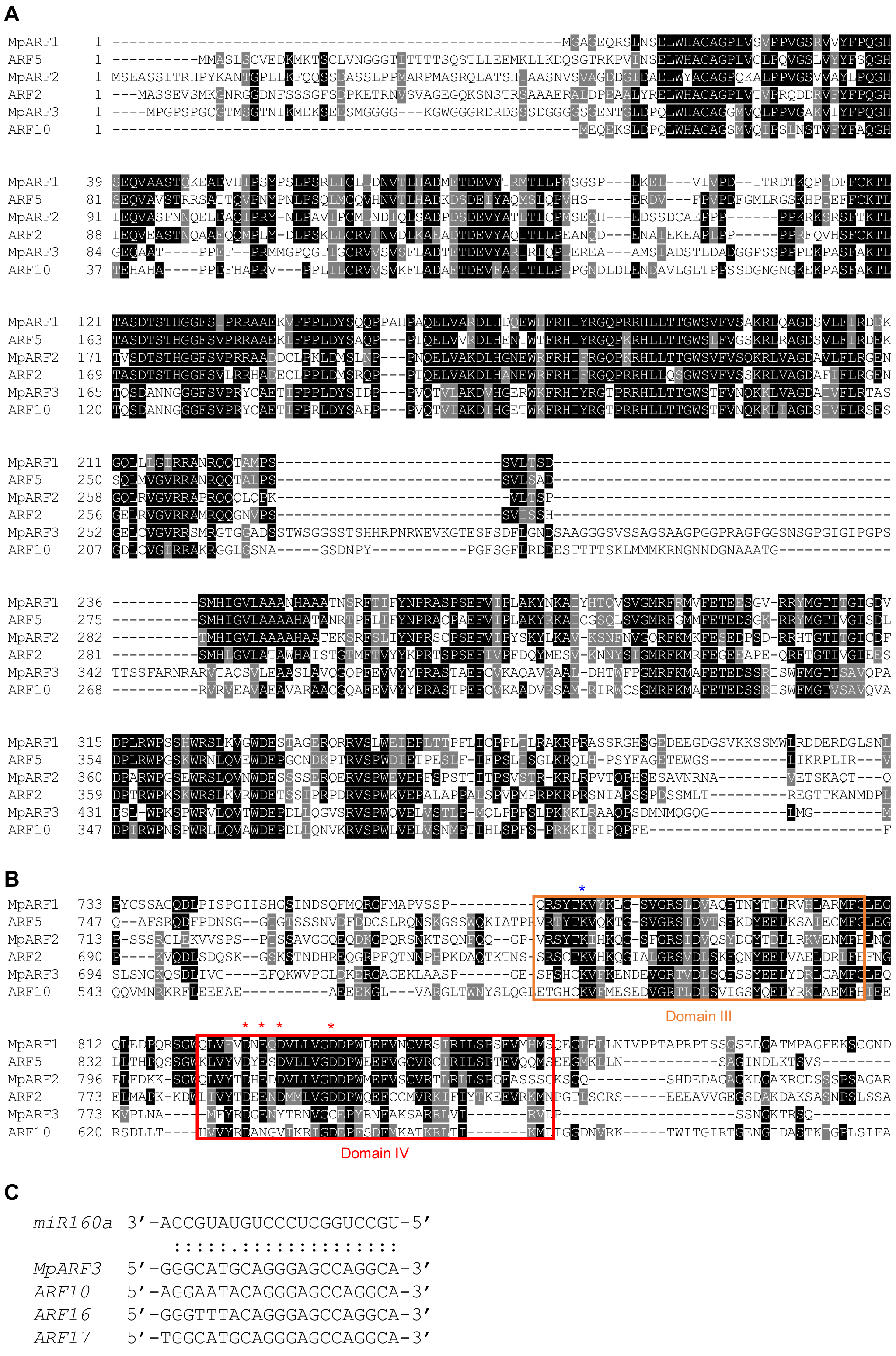

Supplement: S2 Fig — (A, B) Multiple alignment of the DNA-binding domain (A) and C-terminal region (B) of ARF proteins. Protein sequences of ARFs in M. polymorpha and Arabidopsis were aligned using the MUSCLE program. Blue and red asterisks indicate conserved basic and acidic residues, respectively. (C) A possible target site of miR160 in MpARF3 is shown with ARF10, ARF16, ARF17 and miR160a of Arabidopsis. G-C and A-U base pairs are shown as colons, and a G-U base pair is shown as a dot. (TIF) [file pgen.1005084.s002.tif]

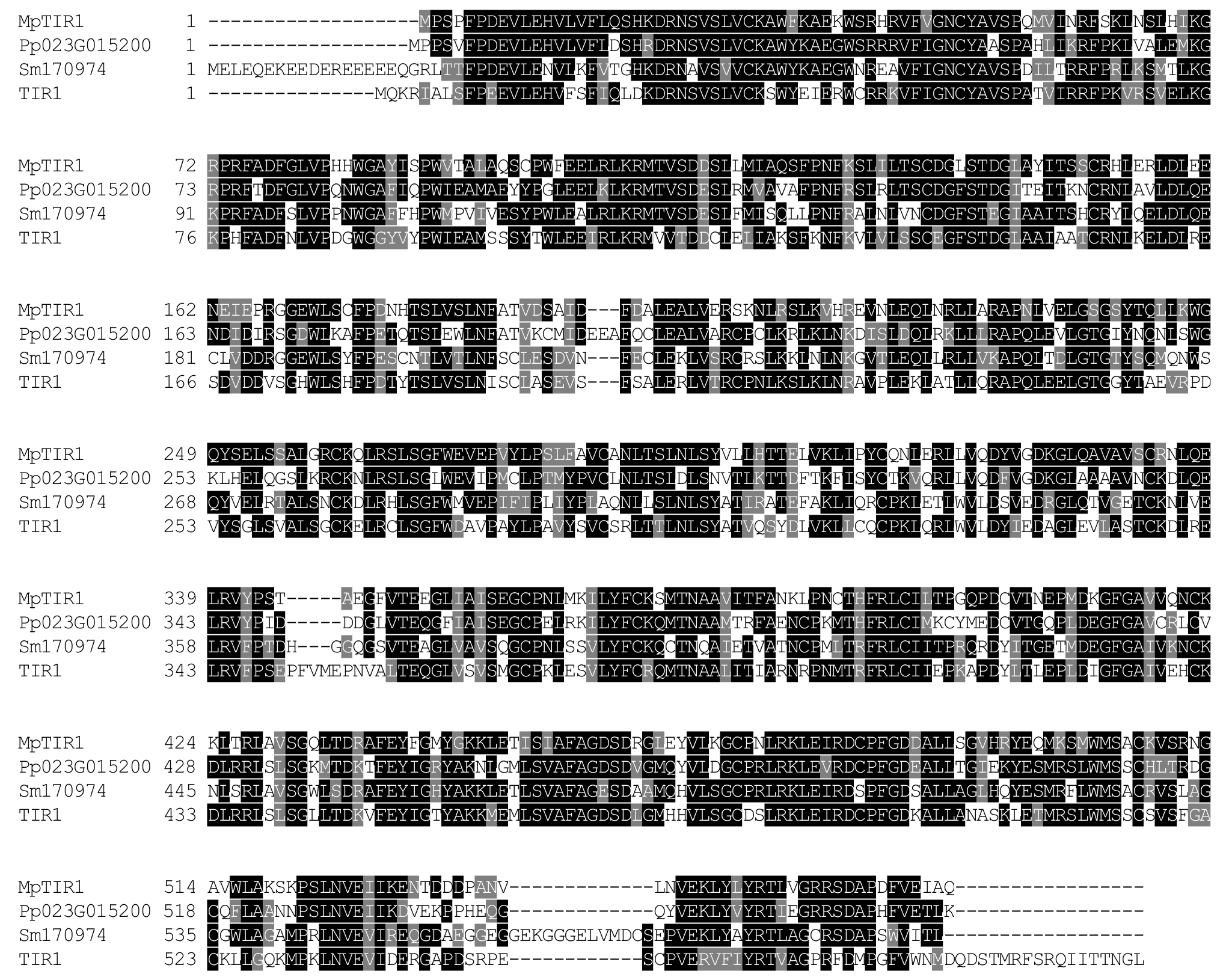

Supplement: S3 Fig — Protein sequences of TIR1/AFBs in M. polymorpha, P. patens, S. moellendorffii and Arabidopsis were aligned using the MUSCLE program. (TIF) [file pgen.1005084.s003.tif]

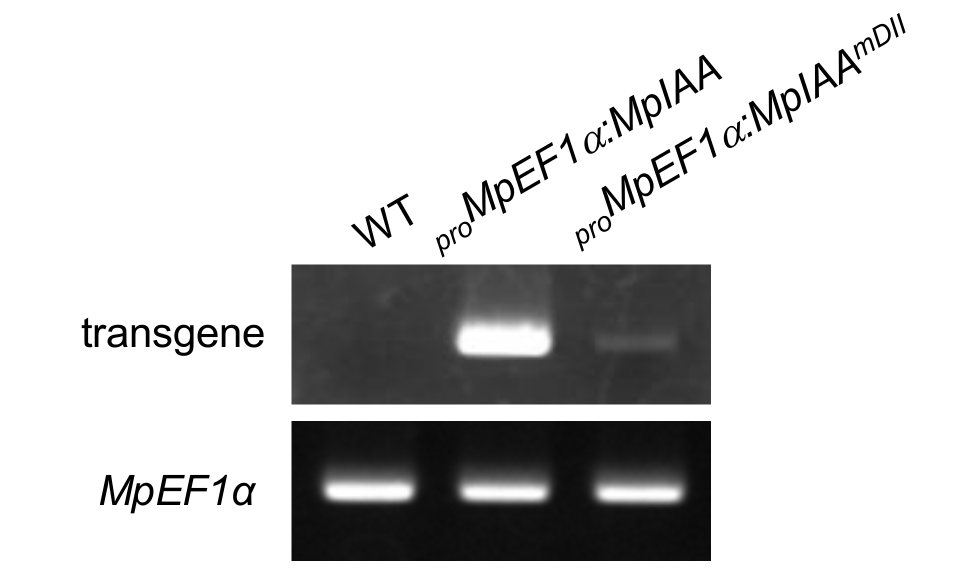

Supplement: S4 Fig — Expression levels of the transgenes in pro MpEF1α:MpIAA and pro MpEF1α:MpIAA mDII plants were analyzed by semi-quantitative RT-PCR using the introduced MpIAA-specific primers. PCR amplification of the cDNA encoding the EF1α was performed and served as a control. WT: wild type. (TIF) [file pgen.1005084.s004.tif]

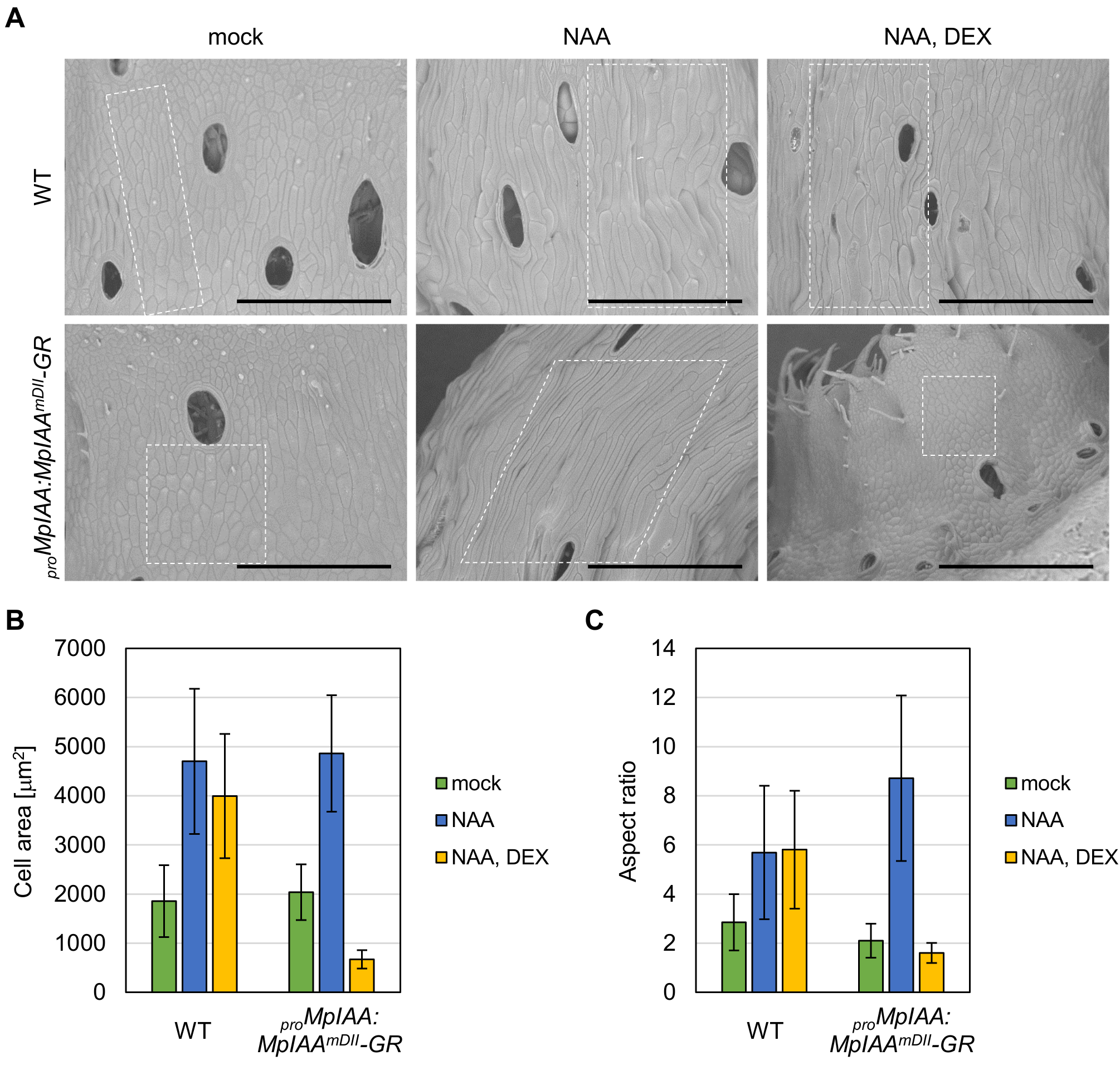

Supplement: S5 Fig — WT and pro MpIAA:MpIAA mDII -GR plants were grown for 12 days in absence of NAA and DEX, then subsequently grown either under the mock condition, with 10 μM NAA, or with 10 μM NAA and 10 μM DEX for 7 days. (A) SEM images of gemma cup. Bars: 0.5 mm. (B-C) The area (B) and the aspect ratio (C) of epidermal cells were measured by imageJ from the SEM images shown in (A). White broken lines indicate measured region. Error bars: SD (n = 50). (TIF) [file pgen.1005084.s005.tif]

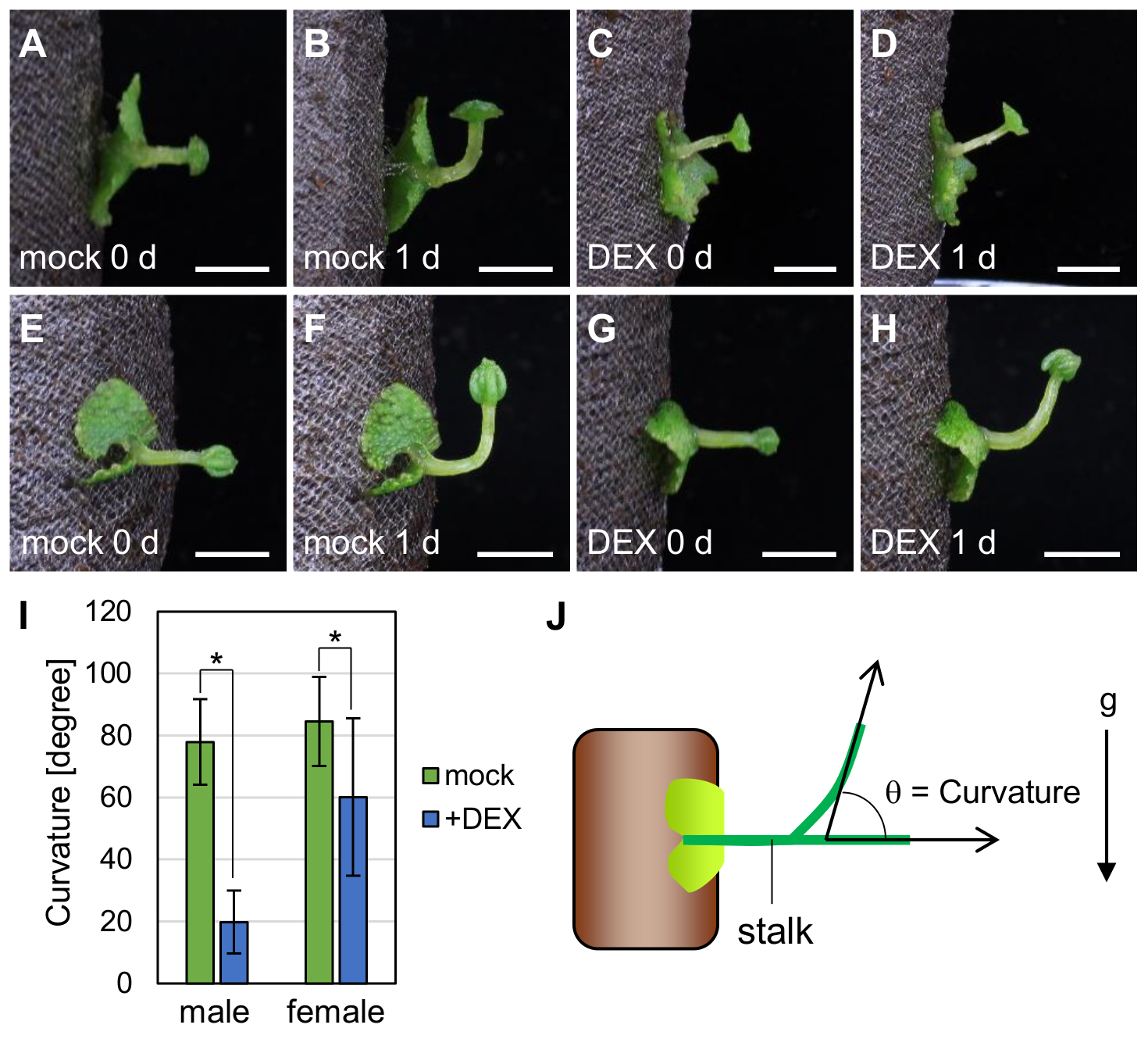

Supplement: S6 Fig — (A-H) Male (A-D) and female (E-H) thalli with visible gametangiophores of pro MpIAA:MpIAA mDII -GR plants were transplanted to new pots (jiffy-7). After 3 days cultivation, water without (A, B, E, F) or with 10 μM DEX (C, D, G, H) was sprayed on the plants. After 3 h, the pots were orthogonally rotated. The images of day 0 (A, C, E, G) and day 1 (B, D, F, H) after rotation were taken. Bars: 5 mm. (I-J) Curvature of gametangiophores was determined as angle between direction of the stalk at day 0 and that at day 1, as shown in (J). Error bars: SD (n≥8). *: P<0.01. g: gravity. (TIF) [file pgen.1005084.s006.tif]
